# Predicting omicron pneumonia severity and outcome: a single-center study in Hangzhou, China

**DOI:** 10.3389/fmed.2023.1192376

**Published:** 2023-05-26

**Authors:** Jingjing Xu, Zhengye Cao, Chunqin Miao, Minming Zhang, Xiaojun Xu

**Affiliations:** ^1^Department of Radiology, The Second Affiliated Hospital, Zhejiang University School of Medicine, Hangzhou, China; ^2^Party and Hospital Administration Office, The Second Affiliated Hospital, Zhejiang University School of Medicine, Hangzhou, China

**Keywords:** artificial intelligence, COVID-19, machine learning, omicron pneumonia, outcome, severity

## Abstract

**Background:**

In December 2022, there was a large Omicron epidemic in Hangzhou, China. Many people were diagnosed with Omicron pneumonia with variable symptom severity and outcome. Computed tomography (CT) imaging has been proven to be an important tool for COVID-19 pneumonia screening and quantification. We hypothesized that CT-based machine learning algorithms can predict disease severity and outcome in Omicron pneumonia, and we compared its performance with the pneumonia severity index (PSI)-related clinical and biological features.

**Methods:**

Our study included 238 patients with the Omicron variant who have been admitted to our hospital in China from 15 December 2022 to 16 January 2023 (the first wave after the dynamic zero-COVID strategy stopped). All patients had a positive real-time polymerase chain reaction (PCR) or lateral flow antigen test for SARS-CoV-2 after vaccination and no previous SARS-CoV-2 infections. We recorded patient baseline information pertaining to demographics, comorbid conditions, vital signs, and available laboratory data. All CT images were processed with a commercial artificial intelligence (AI) algorithm to obtain the volume and percentage of consolidation and infiltration related to Omicron pneumonia. The support vector machine (SVM) model was used to predict the disease severity and outcome.

**Results:**

The receiver operating characteristic (ROC) area under the curve (AUC) of the machine learning classifier using PSI-related features was 0.85 (accuracy = 87.40%, *p* < 0.001) for predicting severity while that using CT-based features was only 0.70 (accuracy = 76.47%, *p* = 0.014). If combined, the AUC was not increased, showing 0.84 (accuracy = 84.03%, *p* < 0.001). Trained on outcome prediction, the classifier reached the AUC of 0.85 using PSI-related features (accuracy = 85.29%, *p* < 0.001), which was higher than using CT-based features (AUC = 0.67, accuracy = 75.21%, *p* < 0.001). If combined, the integrated model showed a slightly higher AUC of 0.86 (accuracy = 86.13%, *p* < 0.001). Oxygen saturation, IL-6, and CT infiltration showed great importance in both predicting severity and outcome.

**Conclusion:**

Our study provided a comprehensive analysis and comparison between baseline chest CT and clinical assessment in disease severity and outcome prediction in Omicron pneumonia. The predictive model accurately predicts the severity and outcome of Omicron infection. Oxygen saturation, IL-6, and infiltration in chest CT were found to be important biomarkers. This approach has the potential to provide frontline physicians with an objective tool to manage Omicron patients more effectively in time-sensitive, stressful, and potentially resource-constrained environments.

## 1. Introduction

The coronavirus disease 2019 (COVID-19) is an ongoing worldwide pandemic. In December 2022, there was a large Omicron epidemic in Hangzhou, China. Despite signs of possibly lower clinical severity than Delta (1), the substantial hospitalizations of Omicron pneumonia had strained the healthcare system in China (2). Notably, the Omicron variant gathered a high number of mutations (3); individuals exhibit significant variability in the severity of presentation and can be re-infected (4, 5). Thus, our understanding of disease manifestation and progression remains unclear. Accurate stratification of the disease severity and outcome is highly desired to effectively handle the pandemic and remains a clinical research priority.

Chest CT is a routine scanning technique for pneumonia, and it plays an important role in COVID-19 infection diagnostics and management (6), especially in the early phase of the pandemic (7). Therefore, CT findings along with clinical and biological biomarkers have been proposed for the prediction of the staging and outcome of COVID-19 pneumonia (8–11). However, data on CT findings of COVID-19 pneumonia originate mainly from early 2020, before the Omicron variants appeared (12). In addition, recent studies have revealed that Omicron, compared with typical Delta, had different CT changes not typical for pneumonia (13–15). As such, the potential of CTs in the Omicron pandemic has not yet been fully realized. Moreover, although some predictors of critical illnesses were shared among these studies, there is currently no consensus as to which clinical variables are most predictive of severity or the need for escalated care. In short, a robust prediction model for the Omicron pneumonia severity and outcome remains lacking.

**Figure 1:**
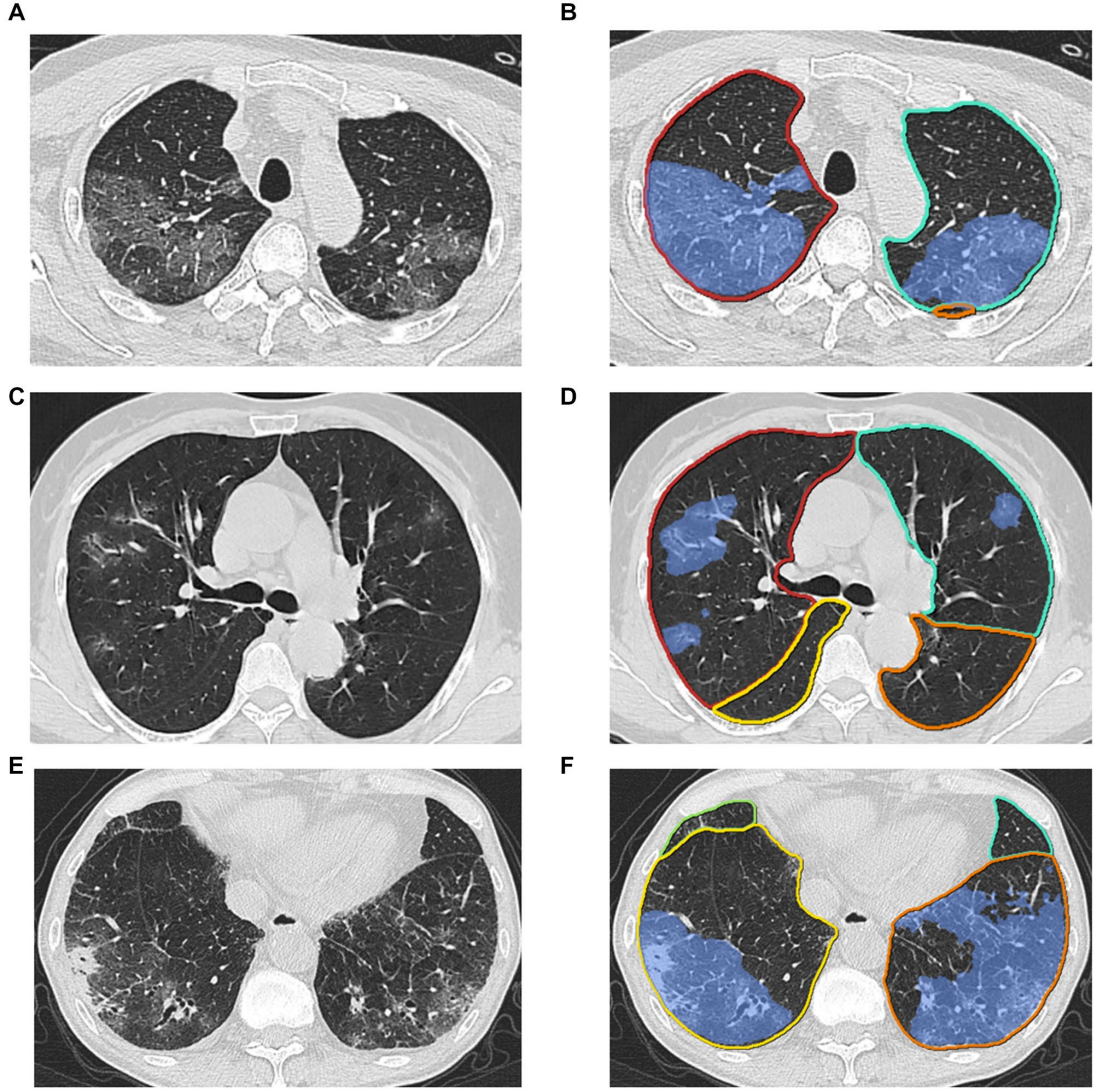
Examples of lesion segmentation by the AI system. Left **(A)**, **(C)**, **(E)**: original images; right **(B)**, **(D)**, **(F)**: pulmonary lobes (colored lines) and opacities segmentation (blue area).

In this study, we investigated an automatic method (Figure 1) of the Omicron pneumonia quantification that extracts image features directly from the CTs and fuses them with known clinical and biological markers. The goal of this study was 2-fold: First, we hypothesized that quantitative image features can be used to predict the severity and clinical outcome of the Omicron pneumonia patients. Second, we hypothesized that the diagnostic power of the presented algorithm using image features is equal to the Pneumonia Severity Index (PSI), serving as the most widely utilized diagnostic model for predicting the prognosis (16). We aim to build predictive models for identifying the severity and outcome of Omicron pneumonia patients at an early stage. Feature importance of both clinical and imaging variables was analyzed to understand the association factors for different disease severity and outcomes. Our goal is to provide early warnings for patients with severe conditions and/or poor outcomes so that doctors could have time to come up with appropriate monitoring and intervention procedures to prevent a worse situation.

## 2. Materials and methods

### 2.1. Patients

Our study included 238 immunocompetent adults with Omicron pneumonia who have been admitted to our hospital in Hangzhou, China, from 15 December 2022 to 16 January 2023. The inclusion criterion was a positive real-time polymerase chain reaction (PCR) or lateral flow antigen test for SARS-CoV-2 after vaccination and no previous SARS-CoV-2 infections. All patients underwent initial laboratory tests and chest CTs. Patients with artifacts and low-quality CTs (incompletely imaged lungs) were excluded.

Omicron pneumonia was clinically classified into non-severe and severe diseases (dyspnea, respiratory frequency over 30/min, oxygen saturation less than 93%, respiratory failure, septic shock, and/or multi-organ dysfunction/failure) (17, 18). The demographic, CT, and clinical characteristics of the patients are presented in Table 1. A binary short clinical outcome was defined as recovered (decreased) and non-recovered (in-hospital death, intubated, and intensive care unit-ICU admission) (19). A total of 238 patients were included, out of which 181 (76.05%) patients had non-severe pneumonia and 57 (23.95%) patients had severe pneumonia, including 146 hospitalization status (61.34%), 57 ICU admissions (23.95%), 34 intubated (14.29%), and 10 death (4.20%) cases. Altogether, our cohort contained a wide range of clinical presentations of Omicron infection with different outcomes.

**Table 1:** Baseline demographic, clinical, and radiological characteristics of adults with radiographic evidence of omicron pneumonia.

Baseline characteristics	All (*n* = 238)	Non severe pneumonia (*n* = 181)	Severe pneumonia (*n* = 57)	*p* value
Clinical parameters				
Age-years (Mean ± SD)	71.84 ± 14.11	69.47 ± 14.32	79.37 ± 10.38	<0.001
Gender (Male/Female)	153/85	113/68	40/17	0.287
Duration from illness onset to hospital presentation-days				
Median	7	7	7	0.640
Interquartile range	4–8.75	5–8	4–10	
Any underlying condition-no. (%)				
Neoplastic disease	31 (13.03)	22 (12.15)	9 (15.79)	0.477
Liver disease	29 (12.18)	25 (13.81)	4 (7.02)	0.171
Chronic heart disease	28 (11.76)	21 (11.60)	7 (12.28)	0.890
Cerebrovascular disease	137 (57.56)	100 (55.25)	37 (62.91)	0.198
Renal disease	37 (15.55)	22 (12.15)	15 (26.32)	0.010
Initial presenting symptoms-no. (%)				
Fever	158 (66.39)	119 (65.74)	39 (68.42)	0.709
Cough	157 (65.97)	119 (65.74)	38 (66.67)	0.898
Chest tightness/pain	76 (31.93)	56 (30.94)	20 (35.09)	0.558
Sputum	72 (30.25)	53 (29.28)	19 (33.33)	0.561
Shortness of breath	53 (22.27)	38 (20.99)	15 (26.32)	0.400
Fatigue/weakness	27 (11.34)	18 (9.94)	9 (15.79)	0.225
Anorexia	17 (7.14)	13 (7.18)	4 (7.02)	0.966
Myalgia	13 (5.46)	7 (3.87)	6 (10.52)	0.054
Sore throat	12 (5.04)	8 (4.42)	4 (7.02)	0.434
Altered mental status	7 (2.94)	3 (1.66)	4 (7.02)	0.037
Time from illness onset to CT-days				
Median	7	7	7	0.776
Interquartile range	5–10	5–10	4–10	
CT findings-no. (%)				
Consolidation	234 (98.32)	177 (97.79)	57 (100)	0.822
Alveolar or interstitial infiltration	167 (70.17)	132 (72.93)	35 (61.40)	0.097
Pleural effusion	89 (37.39)	54 (29.83)	35 (61.40)	<0.001
Pneumonia severity index				
Median	94	87	112	<0.001
Interquartile range	39.5	35	26	
Risk class-no. (%)				
1–3	107 (44.96)	97 (53.59)	10 (17.54)	<0.001
4	105 (44.12)	69 (38.12)	36 (63.16)	
5	26 (10.92)	15 (8.29)	11 (19.30)	

SD: standard deviation, no.: number, comparison of baseline demographic, clinical, and radiological characteristics between the Omicron non-severe pneumonia and severe pneumonia. PSI risk class (no. of points): 1, 2 (≤70), 3 (71–90), 4 (91–130) and 5 (>130) (16).

We reviewed patients’ electronic medical records to obtain information pertaining to their demographics (age, gender), comorbid conditions (such as neoplastic diseases, liver diseases, cardiovascular diseases, chronic heart disease, and renal diseases) (16), baseline vital signs (body temperature, pulse, respiratory rate, and systolic pressure), and baseline laboratory data (including white blood cell count-WBC, C-reactive protein-CRP, blood urea nitrogen-BUN, glucose, sodium, hematocrit, interleukin-6-IL-6, artery pondus hydrogenii-PH, partial pressure of arterial oxygen, and oxygen saturation). We calculated the comorbidity as the score = 5*(0/1, no = 0, yes = 1) as previous studies did (20). Details are presented for further consideration in Table 2. This retrospective study was approved by the ethics committee of the Second Affiliated Hospital of Zhejiang University, School of Medicine.

**Table 2:** Summary of assessed vital signs and lab variables for predicting the need for severity and outcome in omicron pneumonia patients.

Baseline characteristics	All (*n* = 238)	Non severe pneumonia (*n* = 181)	Severe pneumonia (*n* = 57)	*p* value
Vital signs (Mean ± SD)				
Pulse (beats/min)	84.57 ± 13.91	85.01 ± 13.96	83.18 ± 13.78	0.386
Respiratory rate (breaths/min)	18.46 ± 1.60	18.36 ± 1.48	18.75 ± 1.91	0.108
Systolic BP (mmHg)	134.76 ± 18.95	134.03 ± 18.13	137.05 ± 21.36	0.338
Temperature (°C)	37.23 ± 0.76	37.22 ± 0.76	37.28 ± 0.76	0.598
Laboratory data (Mean ± SD)				
BUN (mmol/L)	7.92 ± 5.81	7.51 ± 6.04	9.24 ± 4.81	0.049
Sodium (mmol/L)	138.05 ± 4.99	138.03 ± 4.60	138.10 ± 6.14	0.935
Glucose (mmol/L)	7.68 ± 3.12	7.45 ± 2.92	8.41 ± 3.63	0.071
Hematocrit (%)	36.60 ± 5.30	37.08 ± 4.94	35.10 ± 6.11	0.014
Artery PH	7.41 ± 0.05	7.41 ± 0.04	7.39 ± 0.08	0.118
Partial pressure of arterial oxygen (mmHg)	90.05 ± 25.26	94.43 ± 22.45	76.14 ± 28.08	<0.001
Oxygen saturation (%)	96.04 ± 4.31	97.32 ± 1.42	92.01 ± 7.09	<0.001
CRP (mg/L)	50.33 ± 50.80	45.31 ± 47.92	66.28 ± 56.58	0.006
Total WBC (10^9^/L)	6.35 ± 5.54	6.38 ± 5.34	6.27 ± 6.17	0.895
IL-6 (pg/mL)	66.44 ± 347	26.98 ± 82.20	191.76 ± 685.21	0.075

SD, standard deviation; BUN, blood urea nitrogen; PH, pondus hydrogenii; CRP, C-reactive protein; WBC, white blood cell count; IL, interleukin.

### 2.2. CT image acquisitions

The non-enhanced CT scans were performed using standard clinical parameters with axial 1.5 mm section thickness. All datasets were inspected for quality and excluded in case of incompletely imaged lungs or severe motion artifacts. In detail, the images were acquired on the following scanners: 40 slice scanner (SOMATOM Definition AS) with 120 kV, 65–110 mAs, 1.5 mm slice reconstruction; 64 slice scanner (Philips Brilliance 64) with 120 kV, 160 mAs, 1.5 mm slice reconstruction; and 40 slice scanner (United Imaging uCT 530) with 120 kV, 40–130 mAs, and 1.5 mm slice reconstruction.

### 2.3. CT image evaluations

DICOM images of all chest CTs were imported into a commercial pneumonia AI algorithm (Beijing Deepwise & League of PhD Technology Co.Ltd). The algorithm provides the volume and percentage of consolidation and infiltration area with Omicron-related findings. The processing time per CT was 30–60s. The AI deep learning system: Pytorch 1.1.0, Python 2.7. Operating system: Ubuntu 16.04, Linux. Hardware: Nividia 1080Ti.

The AI algorithm (Supplementary Figure S2) is a deep learning-based model which was built on top of deep convolutional neural networks and proved the performance by previous studies of COVID-19 (21–23). Three major modules were designed to ensure the final accuracy of this system, i.e., pneumonia lesion detection, pneumonia lesion segmentation, and lung lobe segmentation. First, an MVP-Net (24) inspired method was used to detect bounding boxes of pneumonia findings. Channel-wise attention mechanism and multiple inputs (different window centers and window widths) were applied to explore the spatial context of pneumonia, in order to promise the detected sensitivity and multiple symptom classifiers were trained to discriminate consolidation, infiltration, nodules, and so forth. Pneumonia lesions (Figure 1), i.e., voxels that contained pneumonia, were extracted by 3D U-Net (25). Finally, an anatomical prior embedded network was trained to partition the lung into five pulmonary lobes (26).

### 2.4. Features

The PSI-related features contain age, gender, comorbidity, baseline vital signs (body temperature, pulse, respiratory rate, and systolic pressure), and baseline laboratory data (including blood urea nitrogen-BUN, glucose, sodium, hematocrit, artery pondus hydrogenii-PH, partial pressure of arterial oxygen, and oxygen saturation) (16). CT-based features contain consolidation volume, consolidation percentage, infiltration volume, infiltration percentage, total lesion volume, total lesion percentage, and pleural effusion (21–23). Integration features contain baseline laboratory data (including WBC, CRP, and IL-6), all CT-based features, and PSI-related features.

### 2.5. Support vector machine classification

After extracting the desired information from the raw data, a classifier is designed and developed to categorize the severity and outcome of Omicron pneumonia. We applied the support vector machine (SVM) classification (linear kernel was used), a superior method for binary classification, based on these imaging or/and clinical features in Matlab (Mathworks Matlab ver9.2 R2017a, operating system: Microsoft Windows 10.0). The classification problem under consideration discriminates among two mutually exclusive classes (severe or non-severe) (good outcome or poor outcome). Nested 10-fold cross-validation was used in the analysis of the model. A stratified k-fold method was used to divide the data into 10 outer folders, and each outer folder was further subdivided into five inner folders to select the optimal hyperparameter for better training (a grid-search method was used). The predictive performance of each model was examined using accuracy, sensitivity, specificity, and the area under the receiver operating characteristic curve (AUC). We evaluated how well an individual feature contributed to the diagnosis and prognosis prediction, and then, all candidate features were ranked based on their relative importance values. The equation for the relative importance of features is as follows:


$$w=\overset{m}{\underset{i=1}{\sum }}\lambda_{i}y_{i}x_{i}$$

**Figure 2:**
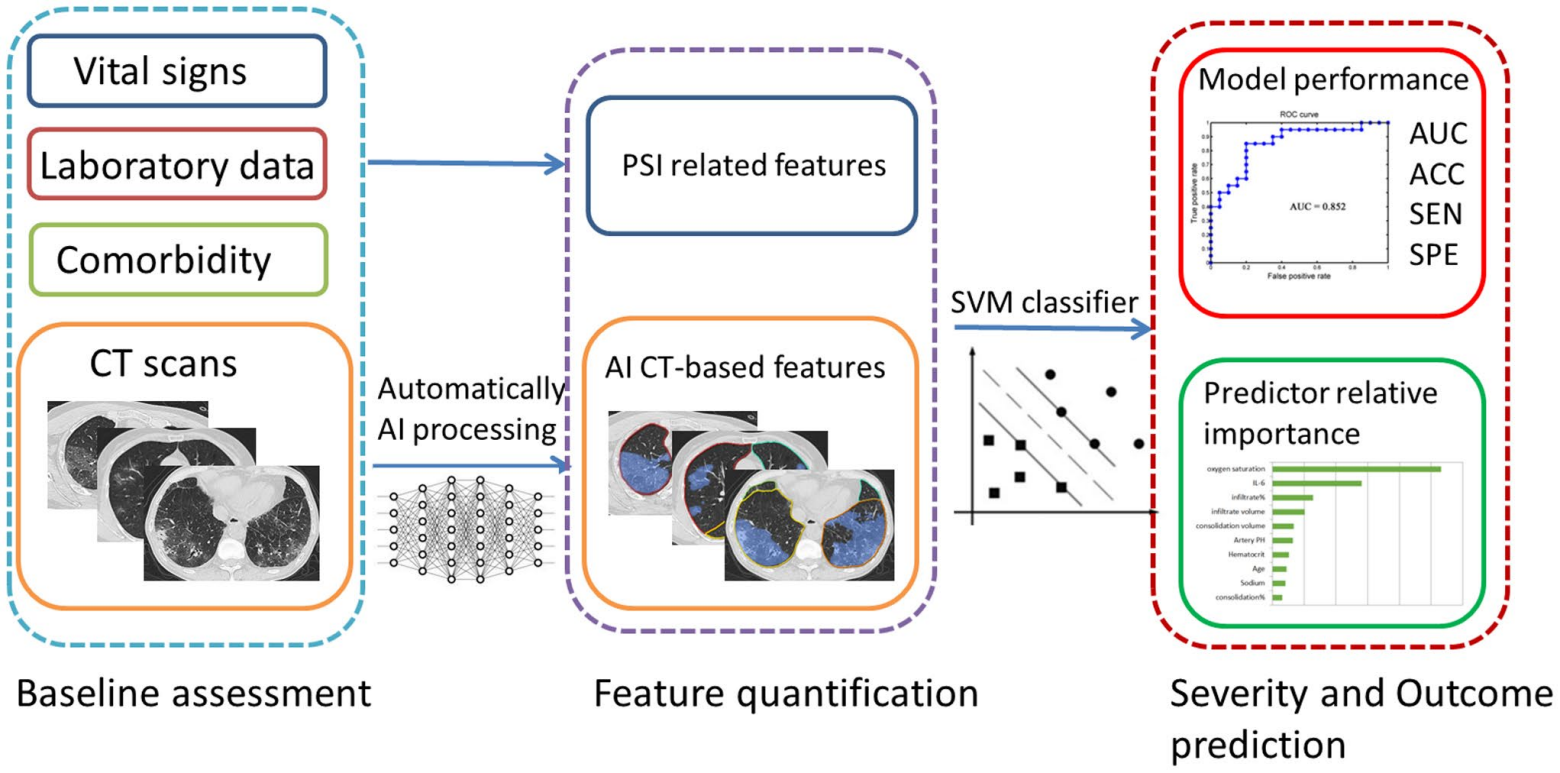
A conceptual overview of the proposed machine learning approach for Omicron pneumonia severity and outcome prediction showing the major processing steps: CT-based image acquisition and segmentation, feature extraction, and statistical learning (SVM). ROC, receiver operating characteristic; AUC, area under the curve; ACC, accuracy; SEN, sensitivity; SPE, specificity.

Statistical significance was evaluated at *p* = 0.05 (permutation test for 1,000 times). We used the SVM algorithms implemented by the Libsvm team (Chih-Chung Chang, Chih-Jen Lin. LIBSVM, a library for support vector machines. 2001). The SVM classifier is available at http://www.csie.ntu.edu.tw/~cjlin/libsvm. Moreover, we provide the packages used for SVM in Supplementary material S1. A conceptual overview of the proposed machine learning approach is presented in Figure 2.

### 2.6. Statistical analyses

Independent *t*-test and chi-square test were used to analyze the quantitative and categorical variables, respectively. IBM SPSS version 19.0 was used to perform all statistical analyses. A two-tailed value of *p* of less than 0.05 was considered to be statistically significant (corrected for multiple comparisons with Bonferroni).

## 3. Results

### 3.1. Patients

Our study included CT images of 238 patients with Omicron pneumonia. Fever is the most commonly reported finding in 66.39% of our patients (65.74% non-severe vs. 68.42% severe), but fever alone does not distinguish the severity. Altered mental status had been emerged as an initial symptom in some of our cases (1.66% non-severe vs. 7.02% severe), which was associated with severe pneumonia (*p* = 0.037). Chest CT findings include consolidation, infiltration, or/and pleural effusions.

A total of 181 (76.05%) patients had non-severe pneumonia and 57 (23.95%) patients had severe pneumonia. Patients with severe Omicron pneumonia had a significantly higher age (*p* < 0.001), higher blood urea nitrogen (*p* = 0.049), higher CRP (*p* = 0.006), lower hematocrit (*p* = 0.014), lower partial pressure of arterial oxygen (*p* < 0.001), lower oxygen saturation (*p* < 0.001), higher CT consolidation volume (*p* = 0.003), higher consolidation percentage (*p* < 0.001), higher infiltration percentage (*p* = 0.031), higher total lesion volume (*p* = 0.001), higher total lesion percentage (*p* < 0.001), and more cases with pleural effusion (*p* < 0.001).

In total, 178 patients (74.79%) had a good outcome and 60 patients (25.21%) had a poor outcome. Patients with poor outcomes had a significantly higher age (*p* = 0.009), higher blood glucose (*p* < 0.001), higher CRP (*p* = 0.002), lower oxygen saturation (*p* = 0.018), and more cases with pleural effusion (*p* = 0.001). The assessed baseline variables including CT features for prediction of severity and outcome are presented in Tables 3, 4.

**Table 3:** Summary of assessed variables for prediction of non-severe versus severe omicron pneumonia in patients.

	Non severe pneumonia (*n* = 181)	Severe pneumonia (*n* = 57)	*p* value
Age-years (Mean ± SD)	69.47 ± 14.32	79.37 ± 10.38	<0.001
Gender (Male/Female)	113/68	40/17	0.287
Comorbidity	1.18 ± 0.91	1.14 ± 0.99	0.796
Pulse (beats/min)	85.01 ± 13.96	83.18 ± 13.78	0.386
Respiratory rate (breaths/min)	18.36 ± 1.48	18.75 ± 1.91	0.108
Systolic BP (mmHg)	134.03 ± 18.13	137.05 ± 21.36	0.338
Temperature (°C)	37.22 ± 0.76	37.28 ± 0.76	0.598
BUN (mmol/L)	7.51 ± 6.04	9.24 ± 4.81	0.049
Sodium (mmol/L)	138.03 ± 4.60	138.10 ± 6.14	0.935
Glucose (mmol/L)	7.45 ± 2.92	8.41 ± 3.63	0.071
Hematocrit (%)	37.08 ± 4.94	35.10 ± 6.11	0.014
Artery PH	7.41 ± 0.04	7.39 ± 0.08	0.118
Partial pressure of arterial oxygen (mmHg)	94.43 ± 22.45	76.14 ± 28.08	<0.001
Oxygen saturation (%)	97.32 ± 1.42	92.01 ± 7.09	<0.001
CRP (mg/L)	45.31 ± 47.92	66.28 ± 56.58	0.006
Total WBC (10^9^/L)	6.38 ± 5.34	6.27 ± 6.17	0.895
IL-6 (pg/mL)	26.98 ± 82.20	191.76 ± 685.21	0.075
AI CT-based features			
Consolidation volume (cm^3^)	328.87 ± 414.73	560.30 ± 510.78	0.003
Consolidation percentage (%)	12.55 ± 13.58	20.36 ± 16.67	<0.001
Infiltration volume (cm^3^)	41.04 ± 211.83	115.99 ± 341.36	0.122
Infiltration percentage (%)	1.30 ± 4.81	4.85 ± 11.80	0.031
Total lesion volume (cm^3^)	369.89 ± 455.74	676.30 ± 637.88	0.001
Total lesion percentage (%)	13.83 ± 14.06	25.19 ± 17.70	<0.001
Pleural effusion (Y/N)	54/127	35/22	<0.001

SD, standard deviation; BUN, blood urea nitrogen; PH, pondus hydrogenii; CRP, C-reactive protein; WBC, white blood cell count; IL, interleukin. Percentage: lesion volume/total lung volume, Y/N: present or absent.

**Table 4:** Summary of assessed variables for prediction of good versus poor outcomes from omicron pneumonia in patients.

	Good outcome (*n* = 178)	Poor outcome (*n* = 60)	*p* value
Age-years (Mean ± SD)	70.46 ± 14.53	75.93 ± 11.98	0.009
Gender (Male/Female)	112/66	41/19	0.449
Comorbidity	1.17 ± 0.91	1.15 ± 0.97	0.862
Pulse (beats/min)	83.43 ± 12.93	87.95 ± 16.12	0.052
Respiratory rate (breaths/min)	18.44 ± 1.61	18.52 ± 1.57	0.743
Systolic BP (mmHg)	134.44 ± 17.41	135.68 ± 23.07	0.704
Temperature (°C)	37.23 ± 0.78	37.23 ± 0.71	0.992
BUN (mmol/L)	7.79 ± 5.98	8.32 ± 5.29	0.539
Sodium (mmol/L)	138.11 ± 4.70	137.85 ± 5.82	0.727
Glucose (mmol/L)	7.15 ± 2.61	9.23 ± 3.93	<0.001
Hematocrit (%)	36.90 ± 4.97	35.72 ± 6.14	0.138
Artery PH	7.41 ± 0.04	7.40 ± 0.07	0.495
Partial pressure of arterial oxygen (mmHg)	90.65 ± 22.15	88.28 ± 32.99	0.606
Oxygen saturation (%)	96.43 ± 4.27	94.90 ± 4.24	0.018
CRP (mg/L)	44.43 ± 47.78	67.83 ± 55.69	0.002
Total WBC (10^9^/L)	6.35 ± 6.08	6.36 ± 3.51	0.984
IL-6 (pg/mL)	22.90 ± 64.74	195.62 ± 671.52	0.051
AI CT-based features			
Consolidation volume (cm^3^)	361.06 ± 448.37	453.28 ± 449.61	0.170
Consolidation percentage (%)	13.51 ± 14.81	17.13 ± 14.26	0.101
Infiltration volume (cm^3^)	63.50 ± 263.56	45.61 ± 207.36	0.633
Infiltration percentage (%)	2.22 ± 7.43	1.96 ± 6.81	0.813
Total lesion volume (cm^3^)	424.53 ± 524.50	498.89 ± 509.24	0.340
Total lesion percentage (%)	15.71 ± 15.86	19.07 ± 15.24	0.153
Pleural effusion (Y/N)	56/122	33/27	0.001

SD, standard deviation; BUN, blood urea nitrogen; PH, pondus hydrogenii; CRP, C-reactive protein; WBC, white blood cell count; IL, interleukin. Percentage: lesion volume/total lung volume, Y/N: present or absent.

### 3.2. Imaging-based severity prediction

**Figure 3:**
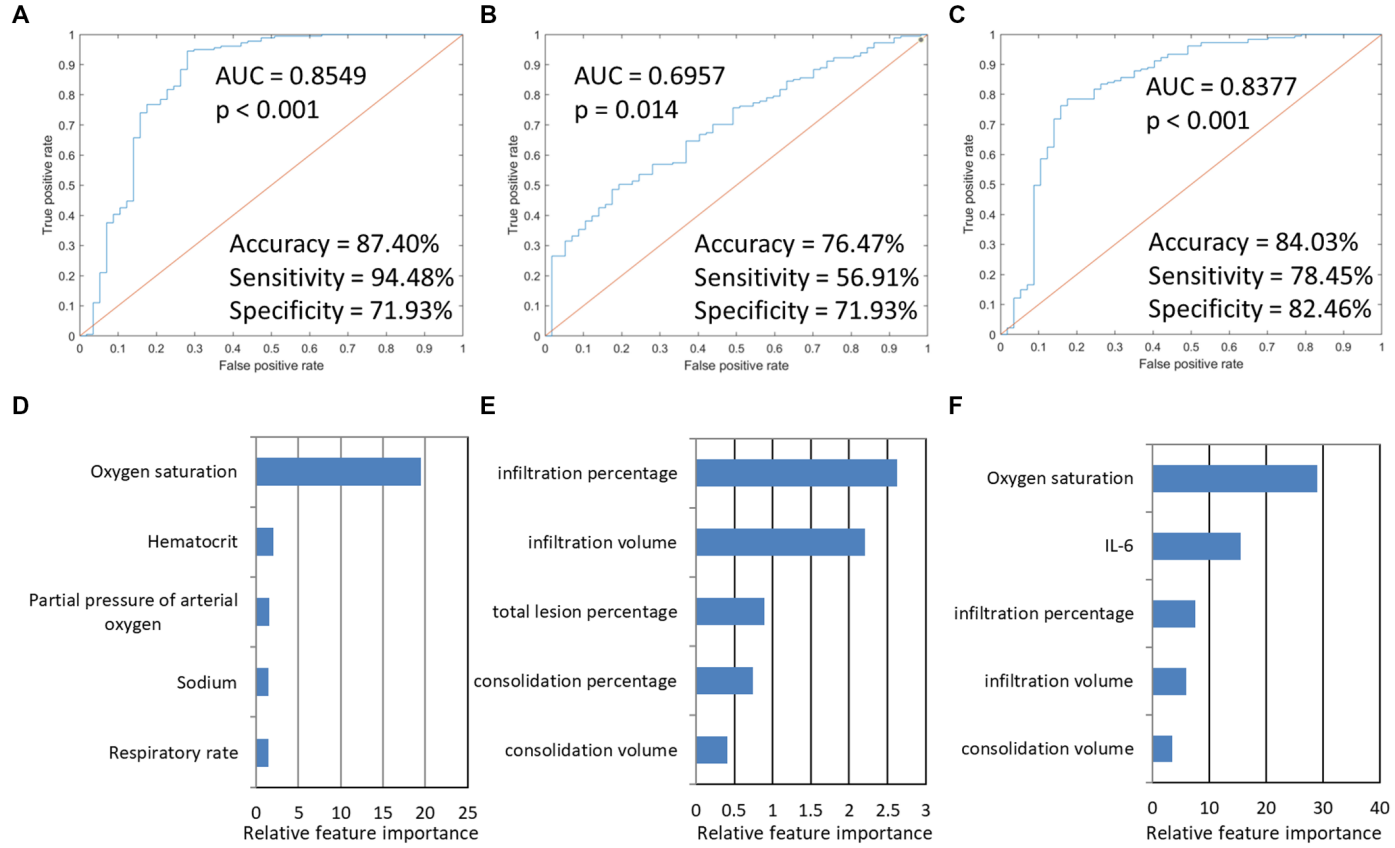
The model performances in the prediction of severity of Omicron pneumonia and the five most important features in the three severity prediction tasks. The first row presented ROC curves for predicting the severity of models based on different data types. **(A)** indicated that PSI-based models for predicting severity achieved the highest AUC (0.8549). **(B)** imaging-based model. **(C)** integration model. **(D–F)** showed the five most important features and their relative importance.

We conducted predictive modeling of Omicron pneumonia diagnosis using the described data. We evaluated and compared the performance of the Imaging-based model, PSI-based model, and integration model. The PSI-based model reached a ROC AUC of 0.85 (accuracy = 87.40%, sensitivity = 94.48%, specificity = 71.93%, *p* < 0.001), which was higher than the purely imaging-based classifier with ROC AUC of 0.70 (accuracy = 76.47%, sensitivity = 56.91%, specificity = 71.93%, *p* = 0.014). If combined, the integrated model showed an equivalent ROC AUC of 0.84 (accuracy = 84.03%, sensitivity = 78.45%, specificity = 82.46%, *p* < 0.001). The predictive performance of each of the three models and the five most important features are presented in Figure 3. For the prediction of severity, the PSI-related features and integrated features had excellent performance. Oxygen saturation, IL-6, and CT infiltration percentage were very important biomarkers. Metrics for the different models studied are presented in Table 5.

**Table 5:** Metrics for the different models studied.

Mean ± SD	AUC	Accuracy	Sensitivity	Specificity
Severity prediction
PSI based model	0.85 ± 0.10	0.87 ± 0.05	0.94 ± 0.10	0.72 ± 0.14
Imaging based model	0.70 ± 0.17	0.76 ± 0.07	0.57 ± 0.15	0.72 ± 0.20
Integrated model	0.84 ± 0.14	0.84 ± 0.06	0.78 ± 0.14	0.82 ± 0.16
Outcome prediction
PSI based model	0.87 ± 0.10	0.85 ± 0.05	0.95 ± 0.10	0.72 ± 0.14
Imaging based model	0.67 ± 0.17	0.76 ± 0.07	0.73 ± 0.15	0.63 ± 0.20
Integrated model	0.84 ± 0.14	0.84 ± 0.06	0.78 ± 0.14	0.82 ± 0.16

SD, standard deviation.

### 3.3. Imaging-based outcome prediction

**Figure 4:**
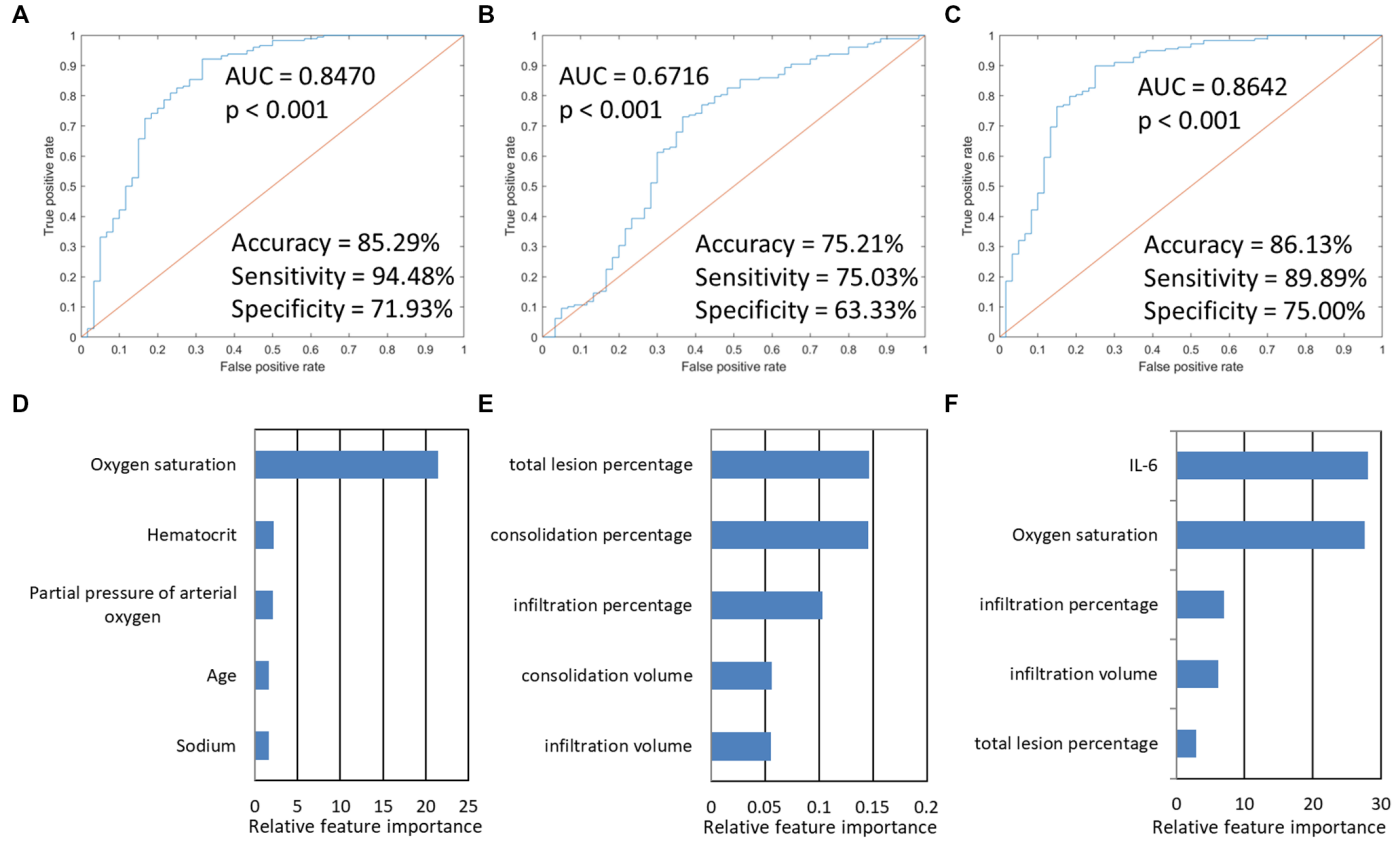
The model performances in the prediction of the outcome of Omicron pneumonia and the five most important features in the three outcome prediction tasks. The first row presented ROC curves for predicting the outcome of models based on different data types. **(A)** PSI-based model. **(B)** imaging-based model. **(C)** indicated that integration models for predicting the outcome achieved the highest AUC (0.8642). **(D–F)** showed the five most important features and their relative importance.

Next, we used this SVM model to stratify the outcomes of patients. The imaging-based model reached a ROC AUC of 0.67 (accuracy = 75.21%, sensitivity = 75.03%, specificity = 63.33%, *p* < 0.001), which was lower than the PSI-based classifier with ROC AUC of 0.85 (accuracy = 85.29%, sensitivity = 94.48%, specificity = 71.93%, *p* < 0.001). If combined, the integrated model showed a slightly higher ROC AUC of 0.86 (accuracy = 86.13%, sensitivity = 89.89%, specificity = 75.00%, *p* < 0.001). The predictive performance of each of the three models and the five most important features are presented in Figure 4. The results found that the three classifiers efficiently predicted good and poor outcomes. IL-6, oxygen saturation, and CT infiltration percentage were very important biomarkers. Metrics for the different models studied are presented in Table 5.

## 4. Discussion

In this study, we used an SVM machine learning model to predict the severity and outcome of Omicron pneumonia in the first-month breakout after the dynamic zero-COVID strategy was stopped in Hangzhou, China. The features we chose were inspected across the recent COVID-19 literature, finding that most of them have been reported as potential markers of diagnosis and prognosis (27). PSI-related clinical and demographic data were more adequate to differentiate between severe and non-severe diseases. Furthermore, PSI-based model and the integrated model showed a relatively efficient performance to predict the outcome, which had better performance than the CT-based model. In baseline evaluation, Omicron pneumonia patients with high levels of IL-6, low levels of oxygen saturation, and greater CT lung infiltration should be monitored closely to minimize the risk of progression to severe conditions/poor outcomes. The results of this study suggest that the value of early CT imaging for predicting the Omicron disease severity and outcome was limited. Similar to other COVID-19 infections, the Omicron patient’s overall clinical condition should be considered more carefully when deciding whether to offer a chest CT scan (28).

In our cohort, most of the cases were infected via domestic transmission. Fever is the most commonly reported finding in our patients, but the absence of fever is inadequate for screening or treatment decisions. Neither cough, chest tightness, dyspnea nor other symptoms. PSI is the most commonly used comprehensive index to assess the severity and prognosis of community-acquired pneumonia patients (16). A higher PSI score indicates a worse condition and a greater risk of poor outcomes. Studies have shown that PSI was a useful tool to discriminate between survivors and non-survivors of COVID-19 pneumonia (29, 30). In our results, the PSI-based model showed an excellent performance to classify the severity of patients with Omicron pneumonia. PSI-based features, especially oxygen saturation, hematocrit, and partial pressure of arterial oxygen were the three most important factors affecting the severity. During the clinical observation, individuals with oxygen saturation levels of less than 93% and respiratory rate of more than 30 per minute should be considered severe COVID-19 conditions (17, 18). Lower oxygen saturation and respiratory distress can progress to critical illness with hypoxic respiratory failure requiring prolonged ventilatory support. Researchers further observed basal oxygen saturation and partial pressure of arterial oxygen could predict unfavorable evolution in pneumonia (31–34). Similarly, Low levels of hematocrit during admission have been associated with poor prognosis and severe disease (35). Hematocrit is a marker that is strongly correlated with blood hyperviscosity, thrombotic complications, and higher mortality in COVID-19 patients (36). This evidence supports our results that oxygen saturation, hematocrit, and partial pressure of arterial oxygen could be examined as a diagnostic tool in screening for severe Omicron pneumonia.

In our Omicron pneumonia cases, ground-glass opacities (infiltration, 167/238 cases, 70.17%), consolidation (234/238 cases, 98.32%) with ill-defined margins, and air bronchograms, with or without pleural effusions (89/238 cases, 37.39%) were present. As per published studies, the percentages of the occurrence of these manifestations vary widely (6). Most of our patients had multiple lesions. However, “White lung” was not found even in severe patients. This may be due to the relatively short time interval between symptom onset and the CT scan (median 7 days, IQR 4–10 days) (6). The CT lesions frequently presented in the bilateral, peripheral, and posterior distribution. These findings were non-specific and overlapped with other infections, thus, the diagnostic specificity of chest CT imaging for COVID-19 is limited (37, 38).

In line with this, from our machine learning models, the CT imaging-based model had only acceptable discriminatory power in predicting disease severity and outcome. Recently, advancements have been made in using AI in the diagnostic imaging field of COVID-19 pneumonia (39–41). Hou et al. developed and compared different machine learning algorithms to predict the likelihood of ICU admission and mortality in COVID-19 patients. Similar to our results, they found that SpO_2_ was the top predictor of mortality and ICU admission (42). Gao et al. built a mortality prediction model for COVID-19 using clinical information in EHRs on admission. The top-weighted features were D-dimer, SpO_2_, and respiratory rate (43). Although some of the predictors of outcome were shared among these and our studies, there is currently no consensus as to which clinical variables are most predictive of poor outcomes. These differences in findings could be due to different outcome measures, patient cohorts, different hospital environments, and analysis methods employed, among other factors. Recently, the prediction and detection of the Omicron variant brought new issues for researchers. However, for predicting Omicron disease severity and outcome, limited results have been published (Table 6). Gupta et al. (44) combined an Extended Convolutional Neural Network (ECNN) and an Extended Recurrent Neural Network (ERNN) to accurately predict Omicron virus-infected cases automatically using chest CT-scan images. Xu et al. (47) developed an ML model to predict the probability of 7-day and 14-day recovery from the Omicron infection. The results remain inconsistent and controversial, with some reporting a good correlation of CT abnormalities with these clinical outcomes while others did not. Our study confirmed the negative prognostic role played in Omicron pneumonia patients by some of the chest CT and clinical features. However, our study of Omicron pneumonia differed from previous studies in several ways. We employed the SVM model, in contrast to the majority of previous studies, which used logistic regression. Our models identified imaging and clinical predictors that accurately predicted both severity and outcome. We also compared PSI-based and imaging-based model performances. Our study included Omicron pneumonia patients and is among the few studies that described a patient cohort in Hangzhou, China.

**Table 6:** Comparing prediction performance from various studies that used non-invasive measures.

Paper	Models	Patient cohort	Predicted disease	Performance evaluation
Gao et al. (43)	LR, SVM, GBDT, NN	COVID-19, Wuhan, China	Mortality risk	AUC = 0.9621, 0.9760, 0.9246
Hou et al. (42)	RF, Xgboost, SVM, NN	COVID-19, New York	Mortality, ICU admission	AUC = 0.89, 0.79
Gupta et al. (44)	ECNN, ERNN	Omicron, Kaggle and UCI dataset	Omicron infection	AUC = 0.9880
Kim et al. (45)	Multivariable logistic regression	Delta and Omicron, South Korean	Clinical course	Correlation *p* = 0.02
Bao et al. (46)	Multivariate regression	Omicron, Shanghai, China	Mortality	Correlation *p* < 0.05
Xu et al. (47)	DT, SVM, RF, AdaBoost, SMOTEENN	Omicron, Shanghai, China	Duration of recovery	AUC = 0.8975, 0.9353
Jayachandran et al. (48)	The Kaplan–Meier method	Omicron, Kerala, India	Mortality	Correlation *p* < 0.05
Ebell et al. (49)	Logistic regression	Omicron outpatient, Allentown, PA	Hospitalization risk	AUC = 0.85–0.87
Zhu et al. (50)	Multivariate regression, ROC curve analyses	Omicron, Nanjing, China	Pneumonia	AUC = 0.701

LR, logistic regression; SVM, support vector machine; GBDT, gradient boosted decision tree; NN, neural network; RF, random forest; ECNN, extended convolutional neural network; ERNN, extended recurrent neural network; ROC, receiver operating characteristic; DT, decision tree.

Furthermore, in the integrated models, CT imaging features were important factors for predicting disease severity and outcome of Omicron pneumonia (Figures 3F, 4F). These findings were consistent with previous studies. For instance, González et al. (51) found that the lung damage on chest CT scans in severe COVID-19 patients increased significantly, indicating their length of invasive mechanical ventilation during the ICU stays. Chassagnon et al. (8) revealed that imaging biomarkers could predict outcome of the COVID-19 patients using automatic deep learning. According to these findings, a more severe lung injury revealed by CT is associated with more severe conditions and poorer outcomes in COVID-19 patients. Researchers suggest that associations between CT lung injury and inflammatory burden might help to justify this problem (52). Another explanation may be that, in the previous studies, there is an inverse relationship between CT lung injury and oxygen saturation (hypoxia) (31), which has notable prognostic implications. Yazdi et al. (53) identified that baseline laboratory tests, such as CRP, WBC, and oxygen saturation, can predict the CT severity of lung involvement. Although further validation is needed, we propose that these markers are individually associated, but not only specific to COVID-19; however, when these markers are combined, they allow describing some of the processes altered in COVID-19 such as an unregulated immune response, an inflammation burden, and tissue hypoxia.

However, although initial evidence is promising, clinical studies of the usefulness of CT imaging in routine screening and management of patients with COVID-19 are still awaited (54). Reviewers found that chest CT had a clinical utility that was limited, particularly for patients who show no symptoms and patients who are screened early in disease progression (55). CT scan was not indicated in a patient who had mild clinical features unless they are at risk for COVID-19 disease progression (56). The limited role of CT in our study may be due to the following reasons: First, our biased patient cohorts only consist of patients with CT-confirmed Omicron pneumonia. Data on CT findings of COVID-19 pneumonia originate mainly from early 2020 before the Omicron variants appeared (12). Recent studies have revealed that Omicron, compared with typical Delta, had different CT abnormalities (13–15). Omicron CT features were non-specific and overlapped with other infections, so the diagnostic value of chest CT imaging is limited (38). Second, we only include baseline CT images for assessment. Therapeutic strategies for patients were not considered in this study. We speculate that multiple images during treatment instead of a single image could indicate further progression of the disease. Our study is not generalizable to a wider population of individuals infected with SARS-CoV-2.

The multi-organ injury was common in our Omicron pneumonia patients (Table 1). Researchers found that the history of comorbidities was significantly different between the non-survivor and survivor groups in COVID-19 patients (57). They have shown a higher proportion of patients with comorbidities in the non-survivor group (57, 58) and those with more severe diseases (59). Ji et al. (20) found that COVID-19 patients with comorbidities were more likely to progress to severe disease than those without comorbidities. Shen et al. (60) found that mortality was significantly associated with comorbidities (e.g., hypertension, COPD, coronary artery disease, heart failure, and chronic kidney disease) in COVID-19 patients (*p* < 0.05). Some studies found that comorbidity at presentation was an independent high-risk factor for COVID-19 progression and mortality (20, 61). We tried binary logistic regression analysis for prediction, and we found that comorbidity was associated with disease severity [OR (95% CI) =2.778 (1.367 ~ 5.645), *p* = 0.005] and outcome [OR (95% CI) =2.628 (1.338 ~ 5.161), *p* = 0.005], which meant that patients with comorbidities were more likely to progress to severe disease and poor outcome than those without. However, comorbidity did not rank high in our cohort relative to other variables (Figures 2, 3), likely because of the small sample sizes or that the clinical variables were indeed more predictive. Notably, previous studies did not directly compare comorbidities and other clinical variables, and thus it is not known or not well established whether comorbidities are more predictive of severity and outcome relative to other clinical variables. Further studies are warranted.

Several limitations deserve comment. First, the sample size was limited due to restrictions during the first-month epidemic wave. Some patients, especially in the outpatient clinic, had incomplete baseline clinical and laboratory data. Second, our study was a retrospective prediction of patients with known outcomes. We minimized bias by ensuring that the investigators processing the laboratory tests or baseline CTs automated AI algorithm were not aware of the patient outcomes before completing the data collection and image analyses. An expansion of sample size in a prospective study design would certainly contribute to further improving the generalizability of our results. Third, we only use one classifier algorithm. As there is no one-fits-all machine learning algorithm, different classifiers result in different performances. Future studies should focus on evaluating different algorithms and comparing their performance. The final limitation was the missing long-term data (e.g., the outcome at 90 days) as it might offer additional information but was not available for this study.

## 5. Conclusion

Our study provided a comprehensive analysis and comparison between baseline chest CT and clinical assessment in disease severity and outcome prediction in Omicron pneumonia. The predictive model accurately predicts the severity and outcome of Omicron infection. Oxygen saturation, IL-6, and infiltration in chest CT were found to be important biomarkers. This approach has the potential to provide frontline physicians with an objective tool to manage Omicron patients more effectively in time-sensitive, stressful, and potentially resource-constrained environments.

## Data availability statement

The raw data supporting the conclusions of this article will be made available by the authors, without undue reservation.

## Ethics statement

The studies involving human participants were reviewed and approved by the ethics committee of the Second Affiliated Hospital of Zhejiang University, School of Medicine. Written informed consent for participation was not required for this study in accordance with the national legislation and the institutional requirements.

## Author contributions

JX designed the study and wrote the first draft of the manuscript. JX and ZC analyzed the imaging data. CM, MZ, and XX assisted with the study design and interpretation of findings. All authors have contributed to and approved the final manuscript.

## Conflict of interest

The authors declare that the research was conducted in the absence of any commercial or financial relationships that could be construed as a potential conflict of interest.

## Publisher’s note

All claims expressed in this article are solely those of the authors and do not necessarily represent those of their affiliated organizations, or those of the publisher, the editors and the reviewers. Any product that may be evaluated in this article, or claim that may be made by its manufacturer, is not guaranteed or endorsed by the publisher.

## References

[1] Maslo C Friedland R Toubkin M Laubscher A Akaloo T Kama B. Characteristics and outcomes of hospitalized patients in South Africa during the COVID-19 omicron wave compared with previous waves. JAMA. (2022) 327:583–4. doi: 10.1001/jama.2021.24868, PMID: 34967859 PMC8719272

[2] Cai J Deng X Yang J Sun K Liu H Chen Z. Modeling transmission of SARS-CoV-2 omicron in China. Nat Med. (2022) 28:1468–75. doi: 10.1038/s41591-022-01855-7, PMID: 35537471 PMC9307473

[3] Harvey WT Carabelli AM Jackson B Gupta RK Thomson EC Harrison EM. SARS-CoV-2 variants, spike mutations and immune escape. Nat Rev Microbiol. (2021) 19:409–24. doi: 10.1038/s41579-021-00573-0, PMID: 34075212 PMC8167834

[4] Pulliam JRC van Schalkwyk C Govender N von Gottberg A Cohen C Groome MJ. Increased risk of SARS-CoV-2 reinfection associated with emergence of omicron in South Africa. Science. (2022) 376:eabn4947. doi: 10.1126/science.abn4947, PMID: 35289632 PMC8995029

[5] Zhang X Wu S Wu B Yang Q Chen A Li Y. SARS-CoV-2 omicron strain exhibits potent capabilities for immune evasion and viral entrance. Signal Transduct Target Ther. (2021) 6:430. doi: 10.1038/s41392-021-00852-5, PMID: 34921135 PMC8678971

[6] Alsharif W Qurashi A. Effectiveness of COVID-19 diagnosis and management tools: a review. Radiography. (2021) 27:682–7. doi: 10.1016/j.radi.2020.09.010, PMID: 33008761 PMC7505601

[7] Inui S Gonoi W Kurokawa R Nakai Y Watanabe Y Sakurai K. The role of chest imaging in the diagnosis, management, and monitoring of coronavirus disease 2019 (COVID-19) insights. Imaging. (2021) 12:155. doi: 10.1186/s13244-021-01096-1, PMID: 34727257 PMC8561360

[8] Chassagnon G Vakalopoulou M Battistella E Christodoulidis S Hoang-Thi TN Dangeard S. AI-driven quantification, staging and outcome prediction of COVID-19 pneumonia. Med Image Anal. (2021) 67:101860. doi: 10.1016/j.media.2020.101860, PMID: 33171345 PMC7558247

[9] Li K Fang Y Li W Pan C Qin P Zhong Y. CT image visual quantitative evaluation and clinical classification of coronavirus disease (COVID-19). Eur Radiol. (2020) 30:4407–16. doi: 10.1007/s00330-020-06817-6, PMID: 32215691 PMC7095246

[10] Tang N Li D Wang X Sun Z. Abnormal coagulation parameters are associated with poor prognosis in patients with novel coronavirus pneumonia. J Thromb Haemost. (2020) 18:844–7. doi: 10.1111/jth.14768, PMID: 32073213 PMC7166509

[11] Yuan M Yin W Tao Z Tan W Hu Y. Association of radiologic findings with mortality of patients infected with 2019 novel coronavirus in Wuhan, China. PLoS One. (2020) 15:e0230548. doi: 10.1371/journal.pone.0230548, PMID: 32191764 PMC7082074

[12] Simpson S Kay FU Abbara S Bhalla S Chung JH Chung M. Radiological Society of North America expert consensus document on reporting chest CT findings related to COVID-19: endorsed by the Society of Thoracic Radiology, the American College of Radiology, and RSNA. Radiol Cardiothorac Imaging. (2020) 2:e200152. doi: 10.1148/ryct.2020200152, PMID: 33778571 PMC7233447

[13] Askani E Mueller-Peltzer K Madrid J Knoke M Hasic D Bamberg F. Computed tomographic imaging features of COVID-19 pneumonia caused by the Delta (B.1.617.2) and omicron (B.1.1.529) variant in a German nested cohort pilot study group. Tomography. (2022) 8:2435–49. doi: 10.3390/tomography8050202, PMID: 36287801 PMC9607412

[14] Tsakok MT Watson RA Saujani SJ Kong M Xie C Peschl H. Chest CT and hospital outcomes in patients with omicron compared with Delta variant SARS-CoV-2 infection. Radiology. (2022) 306:261–9. doi: 10.1148/radiol.220533 35727150 PMC9272784

[15] Yoon SH Lee JH Kim BN. Chest CT findings in hospitalized patients with SARS-CoV-2: Delta versus omicron variants. Radiology. (2022) 306:252–60. doi: 10.1148/radiol.220676, PMID: 35762887 PMC9272824

[16] Aujesky D Fine MJ. The pneumonia severity index: a decade after the initial derivation and validation. Clin Infect Dis. (2008) 47:S133–9. doi: 10.1086/591394, PMID: 18986279

[17] Raoult D Zumla A Locatelli F Ippolito G Kroemer G. Coronavirus infections: epidemiological, clinical and immunological features and hypotheses. Cell Stress. (2020) 4:66–75. doi: 10.15698/cst2020.04.216, PMID: 32292881 PMC7064018

[18] Umakanthan S Sahu P Ranade AV Bukelo MM Rao JS Abrahao-Machado LF. Origin, transmission, diagnosis and management of coronavirus disease 2019 (COVID-19). Postgrad Med J. (2020) 96:753–8. doi: 10.1136/postgradmedj-2020-138234 32563999 PMC10016932

[19] CDC. Severe outcomes among patients with coronavirus disease 2019 (COVID-19) - United States, February 12-march 16, 2020. Morb Mortal Wkly Rep. (2020) 69:343–6. doi: 10.15585/mmwr.mm6912e2, PMID: 32214079 PMC7725513

[20] Ji M Yuan L Shen W Lv J Li Y Chen J. A predictive model for disease progression in non-severely ill patients with coronavirus disease 2019. Eur Respir J. (2020) 56:2001234. doi: 10.1183/13993003.01234, PMID: 32430433 PMC7241108

[21] Ni Q Sun ZY Qi L Chen W Yang Y Wang L. A deep learning approach to characterize 2019 coronavirus disease (COVID-19) pneumonia in chest CT images. Eur Radiol. (2020) 30:6517–27. doi: 10.1007/s00330-020-07044-9, PMID: 32617690 PMC7331494

[22] Wang YC Luo H Liu S Huang S Zhou Z Yu Q. Dynamic evolution of COVID-19 on chest computed tomography: experience from Jiangsu Province of China. Eur Radiol. (2020) 30:6194–203. doi: 10.1007/s00330-020-06976-6, PMID: 32524223 PMC7283983

[23] Yu Q Wang Y Huang S Liu S Zhou Z Zhang S. Multicenter cohort study demonstrates more consolidation in upper lungs on initial CT increases the risk of adverse clinical outcome in COVID-19 patients. Theranostics. (2020) 10:5641–8. doi: 10.7150/thno.46465, PMID: 32373237 PMC7196305

[24] Li ZH Zhang S Zhang J Huang KQ Wang YZ Yu YZ. MVP-net: multi-view FPN with position-aware attention for deep universal lesion detection. arXiv. (2019). doi: 10.48550/arXiv.1909.04247

[25] Ronneberger O. Fischer P. Brox T. (2015). U-net: convolutional networks for biomedical image segmentation. In International Conference on Medical Image Computing and Computer-Assisted Intervention. 234–241.

[26] Wang X. Q. Zhang Q. Y. Zhou Z. Yu Y. Z. Wang Y. Z. (2020). Evaluating multi-class segmentation errors with anatomical prior. IEEE International Symposium on Biomedical Imaging.

[27] Bazargan M Elahi R Esmaeilzadeh A. OMICRON: virology, immunopathogenesis, and laboratory diagnosis. J Gene Med. (2022) 24:e3435. doi: 10.1002/jgm.3435, PMID: 35726542 PMC9350010

[28] Fatima N Khokhar SA Farooq Ur Rehman RM. Chest CT-scan findings in COVID-19 patients: the relationship between the duration of symptoms and correlation with the oxygen saturation level. J Pak Med Assoc. (2023) 73:60–3. doi: 10.47391/JPMA.5586, PMID: 36842008

[29] Preti C Biza R Novelli L Ghirardi A Conti C Galimberti C. Usefulness of CURB-65, pneumonia severity index and MuLBSTA in predicting COVID-19 mortality. Monaldi Arch Chest Dis. (2022) 92:2054. doi: 10.4081/monaldi.2022.2054, PMID: 35225441

[30] Satici C Demirkol MA Sargin Altunok E Gursoy B Alkan M Kamat S. Performance of pneumonia severity index and CURB-65 in predicting 30-day mortality in patients with COVID-19. Int J Infect Dis. (2020) 98:84–9. doi: 10.1016/j.ijid.2020.06.038, PMID: 32553714 PMC7293841

[31] Aalinezhad M Alikhani F Akbari P Rezaei MH Soleimani S Hakamifard A. Relationship between CT severity score and capillary blood oxygen saturation in patients with COVID-19 infection. Indian J Crit Care Med. (2021) 25:279–83. doi: 10.5005/jp-journals-10071-23752, PMID: 33790507 PMC7991766

[32] Effah CY Miao R Drokow EK Agboyibor C Qiao R Wu Y. Machine learning-assisted prediction of pneumonia based on non-invasive measures. Front Public Health. (2022) 10:938801. doi: 10.3389/fpubh.2022.938801, PMID: 35968461 PMC9371749

[33] Nuevo-Ortega P Reina-Artacho C Dominguez-Moreno F Becerra-Muñoz VM Ruiz-Del-Fresno L Estecha-Foncea MA. Prognosis of COVID-19 pneumonia can be early predicted combining age-adjusted Charlson comorbidity index, CRB score and baseline oxygen saturation. Sci Rep. (2022) 12:2367. doi: 10.1038/s41598-022-06199-3, PMID: 35149742 PMC8837655

[34] Qadir FI Kakamad FH Abdullah IY Abdulla BA Mohammed SH Salih RQ. The relationship between CT severity infections and oxygen saturation in patients infected with COVID-19, a cohort study. Ann Med Surg (Lond). (2022) 76:103439. doi: 10.1016/j.amsu.2022.103439, PMID: 35261765 PMC8891116

[35] Ouyang SM Zhu HQ Xie YN Zou ZS Zuo HM Rao YW. Temporal changes in laboratory markers of survivors and non-survivors of adult inpatients with COVID-19. BMC Infect Dis. (2020) 20:952. doi: 10.1186/s12879-020-05678-0, PMID: 33308159 PMC7729703

[36] Shaik A Chen Q Mar P Kim H Mejia P Pacheco H. Blood hyperviscosity in acute and recent COVID-19 infection. Clin Hemorheol Microcirc. (2022) 82:149–55. doi: 10.3233/CH-221429, PMID: 35466930 PMC9741734

[37] Raptis CA Hammer MM Short RG Shah A Bhalla S Bierhals AJ. Chest CT and coronavirus disease (COVID-19): a critical review of the literature to date. AJR Am J Roentgenol. (2020) 215:839–42. doi: 10.2214/AJR.20.23202, PMID: 32298149

[38] Wiersinga WJ Rhodes A Cheng AC Peacock SJ Prescott HC. Pathophysiology, transmission, diagnosis, and treatment of coronavirus disease 2019 (COVID-19): a review. JAMA. (2020) 324:782–93. doi: 10.1001/jama.2020.12839 32648899

[39] Jin C Chen W Cao Y Xu Z Tan Z Zhang X. Development and evaluation of an artificial intelligence system for COVID-19 diagnosis. Nat Commun. (2020) 11:5088. doi: 10.1038/s41467-020-18685-1, PMID: 33037212 PMC7547659

[40] Li L Qin L Xu Z Yin Y Wang X Kong B. Using artificial intelligence to detect COVID-19 and community-acquired pneumonia based on pulmonary CT: evaluation of the diagnostic accuracy. Radiology. (2020) 296:E65–71. doi: 10.1148/radiol.2020200905, PMID: 32191588 PMC7233473

[41] Shan F Gao Y Wang J Shi W Shi N Han M. Abnormal lung quantification in chest CT images of COVID-19 patients with deep learning and its application to severity prediction. Med Phys. (2021) 48:1633–45. doi: 10.1002/mp.14609, PMID: 33225476 PMC7753662

[42] Hou W Zhao Z Chen A Li H Duong TQ. Machining learning predicts the need for escalated care and mortality in COVID-19 patients from clinical variables. Int J Med Sci. (2021) 18:1739–45. doi: 10.7150/ijms.51235, PMID: 33746590 PMC7976594

[43] Gao Y Cai GY Fang W Li HY Wang SY Chen L. Machine learning based early warning system enables accurate mortality risk prediction for COVID-19. Nat Commun. (2020) 11:5033. doi: 10.1038/s41467-020-18684-2, PMID: 33024092 PMC7538910

[44] Gupta AK Srinivasulu A Hiran KK Sreenivasulu G Rajeyyagari S Subramanyam M. Prediction of omicron virus using combined extended convolutional and recurrent neural networks technique on CT-scan images. Interdiscip Perspect Infect Dis. (2022) 2022:1525615–1. doi: 10.1155/2022/1525615, PMID: 36562006 PMC9763984

[45] Kim MH Nam Y Son NH Heo N Kim B Kang E. Antibody level predicts the clinical course of breakthrough infection of COVID-19 caused by Delta and omicron variants: a prospective cross-sectional study. Open Forum Infect Dis. (2022) 9:262. doi: 10.1093/ofid/ofac262, PMID: 35855960 PMC9129209

[46] Bao WJ Fu SK Zhang H Zhao JL Jin HM Yang XH. Clinical characteristics and short-term mortality of 102 hospitalized hemodialysis patients infected with SARS-CoV-2 omicron BA.2.2.1 variant in Shanghai, China. New Microbes New Infect. (2022) 49-50:101058. doi: 10.1016/j.nmni.2022.101058, PMID: 36447944 PMC9691279

[47] Xu Y Ye W Song Q Shen L Liu Y Guo Y. Using machine learning models to predict the duration of the recovery of COVID-19 patients hospitalized in Fangcang shelter hospital during the omicron BA. 2.2 pandemic. Front Med (Lausanne). (2022) 9:1001801. doi: 10.3389/fmed.2022.1001801, PMID: 36405610 PMC9666500

[48] Jayachandran AK Nelson V Shajahan ME. Chest CT severity score as a predictor of mortality and short-term prognosis in COVID-19. J Family Med Prim Care. (2022) 11:4363–7. doi: 10.4103/jfmpc.jfmpc_209_22, PMID: 36353028 PMC9638539

[49] Ebell MH Hamadani R Kieber-Emmons A. Development and validation of simple risk scores to predict hospitalization in outpatients with COVID-19 including the omicron variant. J Am Board Fam Med. (2022) 35:1058–64. doi: 10.3122/jabfm.2022.220056R1, PMID: 36564190

[50] Zhu K Ma S Chen H Xie J Huang D Fu C. Value of laboratory indicators in predicting pneumonia in symptomatic COVID-19 patients infected with the SARS-CoV-2 omicron variant. Infect Drug Resist. (2023) 16:1159–70. doi: 10.2147/IDR.S397231, PMID: 36879854 PMC9985399

[51] González J Benítez ID Carmona P Santisteve S Monge A Moncusí-Moix A. Pulmonary function and radiologic features in survivors of critical COVID-19: a 3-month prospective cohort. Chest. (2021) 160:187–98. doi: 10.1016/j.chest.2021.02.062, PMID: 33676998 PMC7930807

[52] Besutti G Giorgi Rossi P Ottone M Spaggiari L Canovi S Monelli F. Inflammatory burden and persistent CT lung abnormalities in COVID-19 patients. Sci Rep. (2022) 12:4270. doi: 10.1038/s41598-022-08026-1, PMID: 35277562 PMC8914439

[53] Yazdi NA Ghadery AH Seyed Alinaghi S Jafari F Jafari S Hasannezad M. Predictors of the chest CT score in COVID-19 patients: a cross-sectional study. Virol J. (2021) 18:225. doi: 10.1186/s12985-021-01699-6, PMID: 34794467 PMC8600490

[54] Kanne JP Bai H Bernheim A Chung M Haramati LB Kallmes DF. COVID-19 imaging: what we know now and what remains unknown. Radiology. (2021) 299:E262–79. doi: 10.1148/radiol.2021204522, PMID: 33560192 PMC7879709

[55] Waller JV Kaur P Tucker A Lin KK Diaz MJ Henry TS. Diagnostic tools for coronavirus disease (COVID-19): comparing CT and RT-PCR viral nucleic acid testing. AJR Am J Roentgenol. (2020) 215:834–8. doi: 10.2214/AJR.20.23418, PMID: 32412790

[56] Rubin GD Ryerson CJ Haramati LB Sverzellati N Kanne JP Raoof S. The role of chest imaging in patient management during the COVID-19 pandemic: a multinational consensus statement from the Fleischner society. Chest. (2020) 158:106–16. doi: 10.1016/j.chest.2020.04.003, PMID: 32275978 PMC7138384

[57] Chen A Zhao Z Hou W Singer AJ Li H Duong TQ. Time-to-death longitudinal characterization of clinical variables and longitudinal prediction of mortality in COVID-19 patients: a two-center study. Front Med (Lausanne). (2021) 8:661940. doi: 10.3389/fmed.2021.661940, PMID: 33996864 PMC8116568

[58] Ende VJ Singh G Babatsikos I Hou W Li H Thode HC. Survival of COVID-19 patients with respiratory failure is related to temporal changes in gas exchange and mechanical ventilation. J Intensive Care Med. (2021) 36:1209–16. doi: 10.1177/08850666211033836, PMID: 34397301 PMC8442134

[59] Liang W Guan W Chen R Wang W Li J Xu K. Cancer patients in SARS-CoV-2 infection: a nationwide analysis in China. Lancet Oncol. (2020) 21:335–7. doi: 10.1016/S1470-2045(20)30096-6, PMID: 32066541 PMC7159000

[60] Shen B Hou W Jiang Z Li H Singer AJ Hoshmand-Kochi M. Longitudinal chest X-ray scores and their relations with clinical variables and outcomes in COVID-19 patients. Diagnostics (Basel). (2023) 13:1107. doi: 10.3390/diagnostics13061107, PMID: 36980414 PMC10047384

[61] Lu JQ Musheyev B Peng Q Duong TQ. Neural network analysis of clinical variables predicts escalated care in COVID-19 patients: a retrospective study. PeerJ. (2021) 9:e11205. doi: 10.7717/peerj.11205, PMID: 33976972 PMC8061580

