# Supplementary material for: Predicting omicron pneumonia severity and outcome: a single-center study in Hangzhou, China
Source: Front Med (Lausanne). 2023 May 26;10:1192376. doi: 10.3389/fmed.2023.1192376 (PMC10250627; doi:10.3389/fmed.2023.1192376)
Supplement: Supplementary file 2 [file Data_Sheet_2.docx]

Supplementary Material

Predicting Omicron pneumonia severity and outcome: a single-center study in Hangzhou, China

Jingjing Xu, Zhengye Cao, Chunqin Miao, Minming Zhang, Xiaojun Xu*

*** Correspondence:** Xiaojun Xu: xxjmailbox@zju.edu.cn

# Supplementary Figures and Tables

For more information on Supplementary Material and for details on the different file types accepted, please see [here](https://www.frontiersin.org/guidelines/author-guidelines#supplementary-material).

## Supplementary Figures


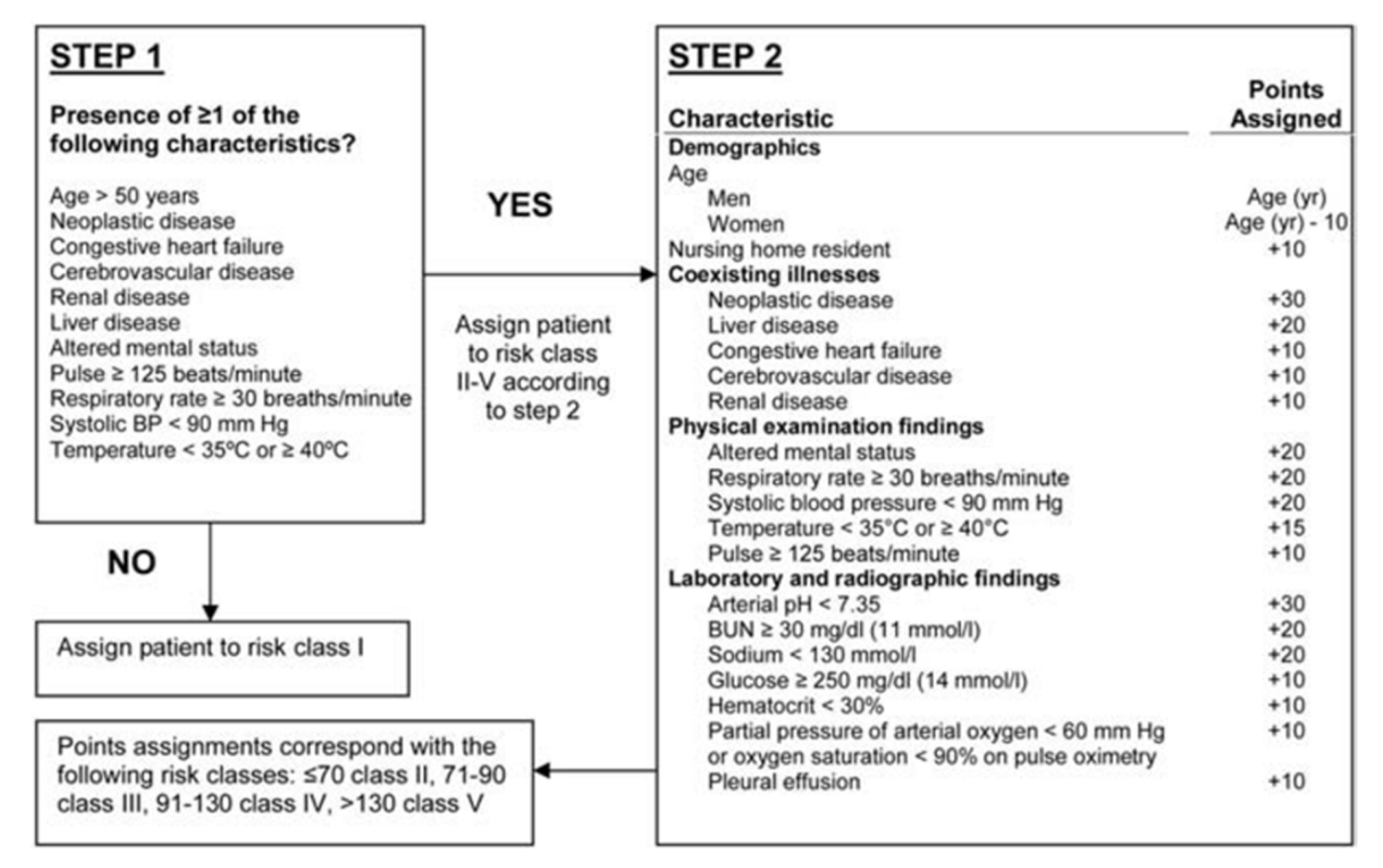


**Supplementary Figure 1.** Assignment to risk class based on the pneumonia severity index. BUN, blood urea nitrogen; yr, years. (Aujesky et al., 2008).


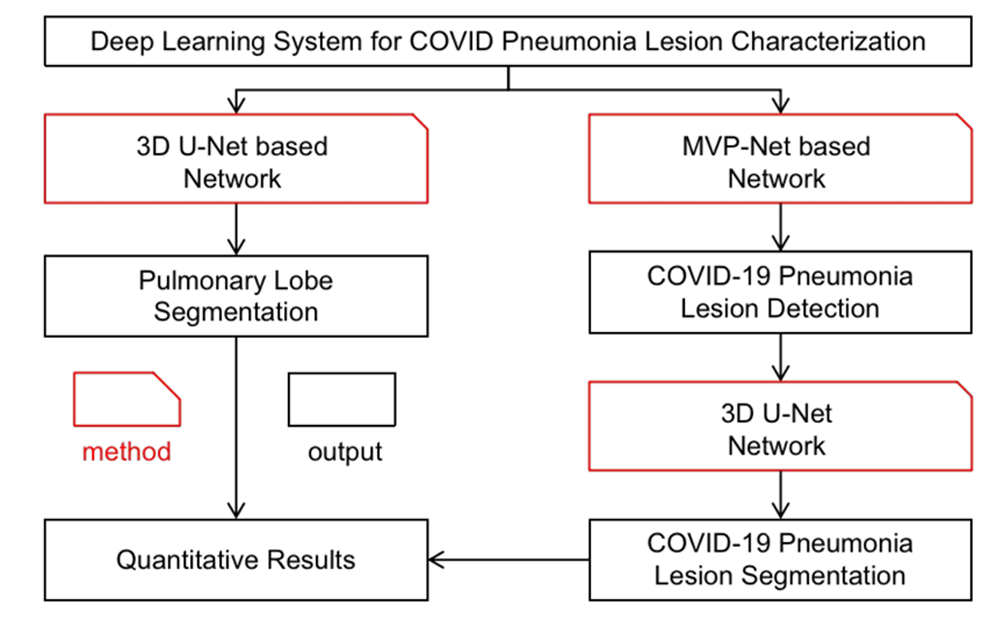


Supplementary Figure 2. Flow chat of the AI system
